# Genomic Survey, Gene Expression Analysis and Structural Modeling Suggest Diverse Roles of DNA Methyltransferases in Legumes

**DOI:** 10.1371/journal.pone.0088947

**Published:** 2014-02-25

**Authors:** Rohini Garg, Romika Kumari, Sneha Tiwari, Shweta Goyal

**Affiliations:** National Institute of Plant Genome Research, Aruna Asaf Ali Marg, New Delhi, India; University of Washington, United States of America

## Abstract

DNA methylation plays a crucial role in development through inheritable gene silencing. Plants possess three types of DNA methyltransferases (MTases), namely Methyltransferase (MET), Chromomethylase (CMT) and Domains Rearranged Methyltransferase (DRM), which maintain methylation at CG, CHG and CHH sites. DNA MTases have not been studied in legumes so far. Here, we report the identification and analysis of putative DNA MTases in five legumes, including chickpea, soybean, pigeonpea, *Medicago* and *Lotus*. MTases in legumes could be classified in known MET, CMT, DRM and DNA nucleotide methyltransferases (DNMT2) subfamilies based on their domain organization. First three MTases represent DNA MTases, whereas DNMT2 represents a transfer RNA (tRNA) MTase. Structural comparison of all the MTases in plants with known MTases in mammalian and plant systems have been reported to assign structural features in context of biological functions of these proteins. The structure analysis clearly specified regions crucial for protein-protein interactions and regions important for nucleosome binding in various domains of CMT and MET proteins. In addition, structural model of DRM suggested that circular permutation of motifs does not have any effect on overall structure of DNA methyltransferase domain. These results provide valuable insights into role of various domains in molecular recognition and should facilitate mechanistic understanding of their function in mediating specific methylation patterns. Further, the comprehensive gene expression analyses of MTases in legumes provided evidence of their role in various developmental processes throughout the plant life cycle and response to various abiotic stresses. Overall, our study will be very helpful in establishing the specific functions of DNA MTases in legumes.

## Introduction

DNA methylation is a conserved epigenetic modification involved in many biological processes. It is responsible for regulation of gene expression patterns and genome stability. It also controls the transcription of invading and mobile DNA elements, such as transgenes, viruses, transposons and retroelements [1], [2]. In plants, DNA is methylated at cytosine residues in three sequence contexts, CG, CHG and CHH (where H is A, C or T) by three types of DNA methyltransferases (MTases), Methyltransferase (MET), Chromomethylase (CMT) and Domains Rearranged Methyltransferase (DRM). MET maintains CG methylation of heterochromatic regions enriched with transposable elements (TEs) and repeats, and genic regions [3], [4]. CMT and DRM mediate CHG and CHH methylation [1], [5]. CMT can initiate DNA methylation *de novo* at sites with certain histone modifications and target silenced transposons and heterochromatin during replication. DRM requires targeting information, which is often derived from small RNA pathway [6].

MTase encoding genes have been identified in several plant species, including *Arabidopsis*, rice, tobacco, maize, wheat and *Physcomitrella* [7]–[11]. Based on phylogenetic analysis, it was proposed that MET and CMT belong to DNMT1 family, whereas DRM belongs to DNMT3 family of MTases. The CMT members appear to be unique to plants [12]. Four and two METs have been identified in *Arabidopsis* and rice, respectively, and three CMTs in each of them [10]. It has been demonstrated that mutation in MET1, results in elimination of CG methylation throughout the genome [3], [4]. Of three members of CMTs identified in *Arabidopsis*, loss of CMT3 results in depletion of CHG methylation, whereas loss of CMT2 results in depletion of CHH methylation in DDM1-dependent manner [13], [14]. DRM1/2 of *Arabidopsis* has been shown to regulate both CHG and CHH methylation in small RNA dependent pathway [13], [14].

Legumes are very important crop plants for human nutrition and their ability to fix atmospheric nitrogen. It is only recently that legumes have gained attention of researchers and the amount of genomic resources available for legumes has been increasing. The draft genome sequence of at least five legumes is available now [15]–[20]. Recently, we reported the draft genome and transcriptome sequences of chickpea for gene discovery [18], [21]. Several genes involved in developmental aspects and stress responses have been identified in legumes, mainly soybean and *Medicago* [17], [22], [23]. However, to our knowledge, no study has reported the analysis of DNA MTases in legumes, so far.

In the present study, we have identified and analyzed DNA MTases in five legumes, *Cicer arietinum* (chickpea), *Glycine max* (soybean), *Cajanus cajan* (pigeonpea), *Medicago truncatula* (*Medicago*) and *Lotus japonicus* (*Lotus*). The phylogenetic relationship among various types of MTases in legumes has been inferred. The gene expression analyses of MTases in various tissues/developmental stages and stress conditions were performed to reveal their putative functions. In addition, three-dimensional (3D) structure modeling of selected members was done along with sequence analysis for identification of the conserved domains and motifs to gain insights into the structure-function conservation. Our analyses provide the framework for future functional studies of this important gene family in legumes.

## Materials and Methods

### Identification of DNA MTases in legumes

DNA MTase proteins in Arabidopsis and rice genome were identified from The Arabidopsis Information Resource (TAIR, http://www.arabidopsis.org/) and Rice Genome Annotation Project (RGAP version 7, http://rice.plantbiology.msu.edu/) databases, respectively, using the keyword DNA methylase and DNA methylase domain (PF00145) search. BLAST search against the annotated protein sequences of chickpea (CGAP v1.0; http://nipgr.res.in/CGAP/home.php) and soybean (Glyma1.1; http://www.phytozome.net/soybean.php) was performed using the DNA MTase protein sequences of rice and Arabidopsis (Table S1 in File S1). In addition, annotated proteome of chickpea and soybean were searched with the hidden Markov model (hmm) profile of Pfam domain PF00145 via HMMER search. The hits obtained by blast and hmm profile searches were filtered using the e-value cutoff of 1e-10 and 1e-5, respectively. The genes identified by both the approaches were combined and redundancy removed. All the proteins were analyzed in SMART (Simple Modular Architecture Research Tool; http://smart.embl-heidelberg.de/) and Pfam databases to confirm the presence of DNA MTase domain. This resulted in identification of a total of 13 and 7 MTases in soybean and chickpea, respectively. Likewise, MTases in other legumes, *Medicago* (Mt3.5; http://www.plantgdb.org/MtGDB/), pigeonpea (PigeonPea_V5.0; http://www.gigadb.org/dataset/100028), *Lotus* (Lj 1.0; http://www.plantgdb.org/LjGDB/), and grapevine (http://www.phytozome.net/grape.php) genome sequences were also identified.

### Sequence analysis

Identification of additional domains in all the identified MTases was performed using SMART search. The motif prediction was done with MEME (Multiple Em for Motif Elicitation, http://meme.nbcr.net/meme/). The presence of the nuclear localization signal in the proteins was analyzed using the tool, cNLS Mapper (http://nls-mapper.iab.keio.ac.jp/cgi-bin/NLS_Mapper_form.cgi).

### Phylogenetic analysis

ClustalW2 (version 2.1) was used to perform the multiple sequence alignment of the identified DNA MTase protein sequences of chickpea, soybean, *Medicago*, pigeonpea, *Lotus*, grapevine, *Arabidopsis* and rice and alignments were visualized using JalView. Phylogenetic trees were generated by the neighbor-joining (NJ) method using Phylogenetic Inference Package (PHYLIP v3.69) with default parameters and displayed using NJ Plot program. Bootstrap analysis was performed with 1000 replicates to obtain a support value for each branch.

### Homology modeling

The templates for homology modeling were selected using the best hit in BLAST searches in the PDB database. Homology model of the protein sequences was generated using Modeller (version 9.11). At least 50 models for each protein were generated using model.py program of Modeller and the best model was selected based on the lowest Discrete Optimized Protein Energy (DOPE) score value. For GmDRM5, advanced modeling was performed using threading method on Protein Homology/analogY Recognition Engine V 2.0 (Phyre2; http://www.sbg.bio.ic.ac.uk/phyre2/), which models protein structure on multiple templates. Model refinements were done using Knowledge-based Potential Refinement for Proteins refinement tool (KoBaMIN; http://csb.stanford.edu/kobamin/). The Ramachandran statistics was calculated by Rampage (http://mordred.bioc.cam.ac.uk/~rapper/rampage.php) for validation of each best selected model.

### Plant material and RNA isolation

We collected 17 tissues, including seven vegetative tissues (root, shoot, mature leaf, young leaf, shoot apical meristem (SAM), germinating seedling and stem), nine stages of flower development from young flower buds to mature flowers (flower buds at sizes 4 mm (FB1), 6 mm (FB2), 8 mm (FB3), 8–10 mm (FB4) and flowers with closed petals (FL1), partially opened petals (FL2), opened petals (FL3), opened and faded petals (FL4) and senescing petals (FL5)), and young pod from chickpea (*Cicer arietinum* genotype ICC4958) plants as described earlier [18], [24]. For abiotic stress treatments, 10-day-old seedlings were subjected to various abiotic stresses as described previously [25]. At least three independent biological replicates of each tissue sample were harvested and immediately frozen in liquid nitrogen. Total RNA from all tissue samples was extracted using TRI reagent (Sigma Life Science, St. Louis, MO) according to manufacturer's instructions. The quality and quantity of RNA was determined using Nanodrop 1000 spectrophotometer (Thermo Fisher Scientific, Wilmington, DE) and Bioanalyzer RNA nano chip (Agilent Technologies, Singapore). The isolated RNA samples were used for the real-time PCR analysis.

### Gene expression analysis

For chickpea, we performed gene expression analysis using RNA-seq data and real-time PCR analysis. The RPKM normalized RNA-seq data for eight tissues/organs, including germinating seedling, root, shoot, stem, young leaf, mature leaf, SAM, young pod and nine stages of flower development [18], [24], were used to study the differential gene expression during chickpea development. The gene expression of chickpea DNA MTases in various tissues/organs was also validated by quantitative real-time PCR analysis. In addition, we performed real-time PCR analysis to study the gene expression of chickpea MTases under various abiotic stress conditions. Briefly, cDNA was synthesized from independent biological replicate RNA sample, and three technical replicates of each biological replicate were analyzed for real-time PCR analysis using SYBR green chemistry employing 7500 Sequence Detection System (Applied Biosystems) as described previously [25]. *EF1α* was used as an internal control gene [25] for normalization of real-time PCR results. Fold change in different tissues was calculated with respect to mature leaf in development series and root and shoot control samples for stress series. Gene-specific primers used are given in Table S2 in File S1.

For soybean, the gene expression data from RNA-seq representing 14 tissues, including young leaf, flower, one cm pod, pod shell (10 days after fertilization, DAF), seed (from 10 DAF to 42 DAF), root and nodule, available at SoyBase (http://soybase.org/soyseq/) were used. In addition, RNA-seq data for nine tissues, including root, root tip, leaves, and root hair cells harvested after 84 h and 120 h after sowing (HAS), nodule, apical meristem, flower, green pod and leaf available at SoyKB (http://www.soykb.org/) was used after RPKM normalization for gene expression analysis. For *Medicago*, gene expression data was obtained from *Medicago truncatula* Gene Expression Atlas (MtGEA, http://mtgea.noble.org/v3/) for 19 tissues representing, leaf, petiole, vegetative bud, stem, flower, pod and root from 28-day-old plant alongwith different stages of seed development and nodulation [22]. For *Lotus*, gene expression data from *Lotus japonicus* Gene Expression Atlas (LjGEA, http://ljgea.noble.org/v2/) for 14 tissues representing, leaf, petiole, stem, root, from 28 day old plant along with fully opened flower and nodule 21 days post infection together with different stages of pod and seed development was used for expression analysis [26]. The heatmaps were plotted using MultiExperiment Viewer (v4.8.1).

### Subcellular localization

Full-length cDNAs of selected MTases identified in chickpea were amplified by reverse transcription-PCR using total RNA isolated from flower bud using gene-specific primers and cloned in pGEM-T easy vector (Promega). The complete (*CaCMT1*, 2844 bp and *CaDRM1*, 1818 bp) or N-terminal (N-terminal 1122 bp of *CaMET1*) coding regions of CaMTases were amplified from their respective full-length cDNA clones using gene-specific primers (Table S2 in File S1) and fused in-frame, downstream to GFP in psGFPcs vector and bombarded onto onion epidermal cells using a particle gun (Bio-Rad) and visualized under a fluorescence microscope (AOBS TCS-SP2, Leica Microsystems) after 24 h as described earlier [27].

## Results

### Identification and classification of DNA MTases in legumes

**Figure 1:**
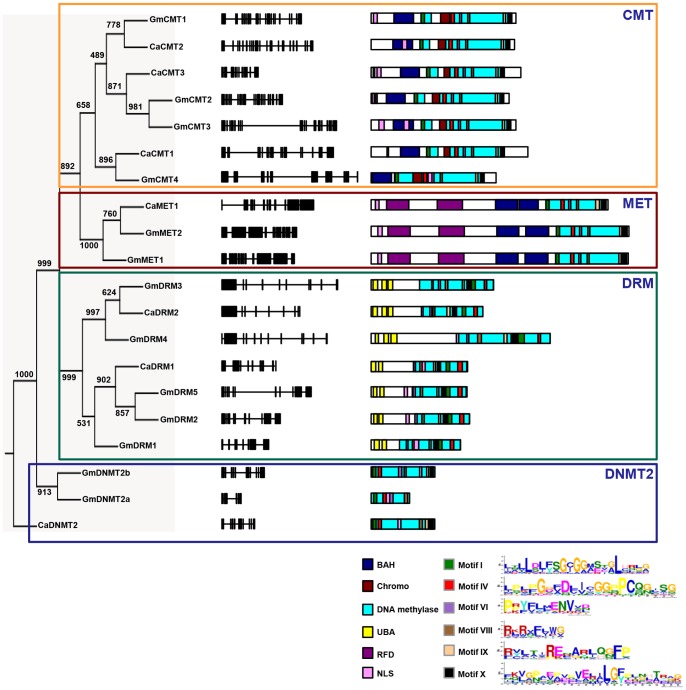
Phylogenetic analysis, and gene and protein structure of methyltransferases (MTases) in chickpea and soybean. Phylogenetic tree of MTases of chickpea and soybean (*left panel*). The numbers at the nodes represent bootstrap values from 1000 replicates. Intron-exon organization of chickpea and soybean MTase genes is shown in *middle panel*. Exons are shown as black boxes and introns are represented by lines. The domain and motif organization in chickpea and soybean MTases are shown in the *right panel*. Different domains and motifs are shown in different colors along with the consensus sequence of predicted motifs as indicated in the legend. *Ca,* chickpea; *Gm,* soybean.

**Figure 2:**
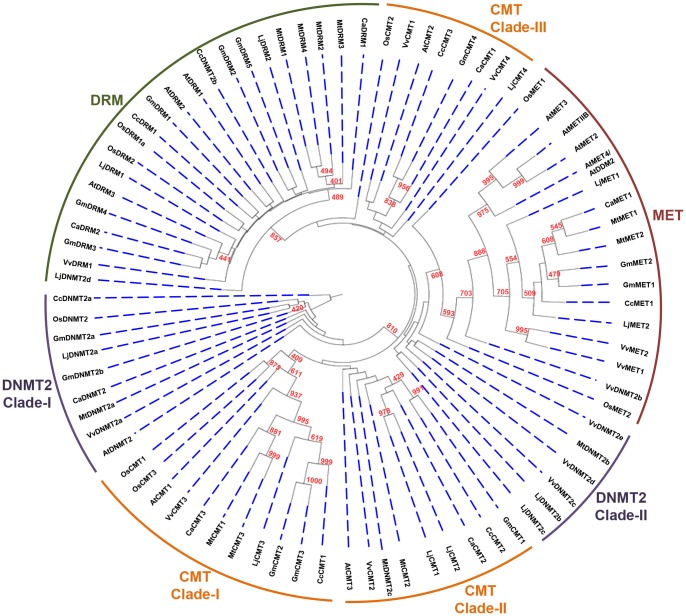
Phylogenetic relationship among various MTases of legumes, *Arabidopsis*, rice and grapevine. An unrooted tree from full-length protein sequences of all the members was constructed. MTases from eight plant species were grouped into different classes, including MET, DRM, CMT and DNMT2. The number at the nodes represents the bootstrap values from 1000 replicates. Soybean (Gm), chickpea (Ca), *Lotus* (Lj), *Medicago* (Mt), pigeonpea (Cc), rice (Os), *Arabidopsis* (At), grapevine (Vv).

Based on BLAST and hmm profile searches followed by domain analysis, a total of 13 and seven MTases were identified in soybean and chickpea, respectively (Table 1). The gene length varied from 2 to 15 kb and 3.6 to 12 kb in soybean and chickpea, respectively (Table 1). The protein length varied from 235 to 1555 aa in soybean and 381 to 1494 aa in chickpea (Table 1). In addition, we identified 12 MTases in *Medicago*, seven in pigeonpea and 12 each in *Lotus* (Table S3 in File S1). We also identified 12 MTases in *Vitis vinifera* (grapevine) to use as an outgroup for various analyses (Table S3 in File S1). Based on the presence of amino-terminus domains, such as ubiquitin-associated domain (UBA), bromo adjacent homology (BAH) domain, chromodomain (Chr) and replication foci domain (RFD), MTases were grouped into four subfamilies. MTases containing RFD, BAH and methyltransferase domains were classified as MET family members, whereas members with Chr domain along with BAH and methyltransferase domain were placed in CMT family (Figure 1). Members harboring both UBA and methyltransferase domains were grouped into DRM family (Figure 1). As compared to MET, CMT and DRM classes, DNA nucleotide methyltransferases (DNMT2) class members appear to lack any amino-terminal regulatory domain and contain only methyltransferase domain (Figure 1). In soybean, a total of four genes were identified as CMT, two as MET, five as DRMs and two as DNMT2 members, whereas in chickpea three members belonged to CMT, one to MET, two to DRM and one to DNMT2 family (Figure 1). Similarly, we also identified members of different families in *Medicago* (three CMTs, two METs, four DRMs and three DNMT2s), *Lotus* (four CMTs, two METs, two DRMs and four DNMT2s), pigeonpea (three CMTs, one MET, one DRM and two DNMT2s) and grapevine (four CMTs, two METs, one DRM and five DNMT2s) (Figure 2; Table S3 in File S1). The intron-exon organization (number of introns/exons) was found conserved between members of MTase families in soybean and chickpea (Table 1). The coding region of CMT genes was interrupted by 18–20 introns. Similar numbers of introns have been reported in *Arabidopsis* CMT genes as well [28]. MET gene length varied from approximately 8 kb in soybean to 10 kb in chickpea harboring 11 introns (Figure 1, middle panel). The length of the DRM genes ranged from 5–12 kb in soybean to about 6–8 kb in chickpea with 9–11 introns. DNMT2 genes were smallest in length harboring 4–9 introns.

**Table 1:** Methyltransferases identified in chickpea and soybean.

Gene name	Gene identifier	Genomic location (chromosome/scaffold)	Gene length (bp)	No. of introns/exons	Protein length (aa)
Chickpea					
*CaCMT1*	Ca_07678	CaLG_7	12129	18/19	947
*CaCMT2*	Ca_03840	CaLG_3	9908	20/21	864
*CaCMT3*	Ca_15245	Scaffold01218	5752	17/18	913
*CaMET1*	Ca_08923	Scaffold00216	10009	11/12	1494
*CaDRM1*	Ca_06704	CaLG_6	5862	9/10	605
*CaDRM2*	Ca_18709	Scaffold03978	8474	8/9	674
*CaDNMT2*	Ca_00204	CaLG_1	3627	9/10	381
Soybean					
*GmCMT1*	Glyma01g01120	Chr1	8654	20/21	875
*GmCMT2*	Glyma01g36500	Chr2	6619	20/21	833
*GmCMT3*	Glyma11g08861	Chr11	12481	20/21	882
*GmCMT4*	Glyma16g17720	Chr16	14726	18/19	754
*GmMET1*	Glyma04g36150	Chr4	7906	11/12	1551
*GmMET2*	Glyma06g18790	Chr6	8148	11/12	1555
*GmDRM1*	Glyma02g04060	Chr2	5149	8/9	537
*GmDRM2*	Glyma05g08740	Chr5	6392	10/11	590
*GmDRM3*	Glyma07g36081	Chr7	12591	9/10	694
*GmDRM4*	Glyma17g04254	Chr17	11453	11/12	738
*GmDRM5*	Glyma19g00250	Chr19	9732	10/11	580
*GmDNMT2a*	Glyma08g18955	Chr8	2151	4/5	235
*GmDNMT2b*	Glyma15g06040	Chr15	4658	9/10	385

### Sequence analysis

The methyltransferase domain contains several conserved motifs required for catalytic transfer of methyl group from S-adenosyl-methionine onto DNA and cytosine methylation [29]. We identified six highly conserved motifs I, IV, VI, VIII, IX and X, present in the methyltransferase domain via MEME analysis in all the 20 MTases from soybean and chickpea (Figure 1) similar to those reported in other plant species (10, 11). Based on X-ray crystallography and multiple sequence alignment, motifs X and I have been identified as S-adenosyl-l-methionine binding subdomains, and motifs, IV, VI, VIII and IX, are the functional catalytic sites in cytosine-5 methyltransferases. It has been suggested that the variable region between motifs VIII and IX (termed as target recognition domain, TRD) determines the sequence specificity of methylation [30]. Each family of MTase was found to have a characteristic arrangement of these motifs in the methyltransferase domain. MET members showed the order of motifs as, I, IV, VI, VIII, IX and X (Figure S1A in File S1). In CMT members, Chr domain was present between the conserved motifs, I and IV, with rest of the arrangement similar to the MET members (Figure S1B in File S1). DRM proteins showed circular permutation of motifs in the cytosine methyltransferase domain with motifs VI through X preceding the motifs I-IV (Figure S1C in File S1). Multiple sequence alignment showed the presence of two or three UBA domains in the DRM family members (Figure S1C in File S1). In *Arabidopsis,*it has been reported that AtDRM2 requires catalytically mutated AtDRM3 for normal establishment and maintenance of RNA-directed DNA methylation and accumulation of specific repeat associated siRNA [31]. Homologues of inactive DRM were identified in chickpea (*CaDRM2*) and soybean (*GmDRM3* and *GmDRM4*) also (Figure S1C in File S1), which lack asparagine-asparagine-leucine (NNL) residues after conserved proline-cysteine in motif IV, glutamic acid in motif IX and glycine in motif X. It will be interesting to analyze, whether they posses methyltransferase activity or not in legumes. DRM3 members have three UBA domains, whereas DRM2 members have two UBA domains. The second UBA domain of catalytically inactive DRM3 members showed substitution of the conserved glycine residue (MGF/MGY) for either lysine or asparagine, which was predicted to abolish proper folding of UBA domain [32]. DNMT2 members of legumes also show conserved cystiene residue in motif IV (PCQ loop) and glutamate residue in motif VI (ENV, Figure S1D in File S1), which is involved in RNA methylation.

We predicted the presence of nuclear localization signal (NLS) in all the MTases of soybean and chickpea (Table S4 in File S1). Both monopartite and bipartite type of NLSs were predicted in different members. For example, monopartite NLS was predicted in CaCMT1, CaCMT3, CaDRM2, CaDNMT2, GmCMT2, GmDRM3 and GmDRM4 proteins and bipartite NLS in CaCMT2, CaCMT3, CaMET1, CaDRM1, GmCMT1, GmCMT2, GmCMT3, GmMET1, GmMET2, GmDRM1, GmDRM2, GmDRM5, GmDNMT2a and GmDNMT2b. Several members showed the presence of more than one bipartite NLS, such as CaCMT2 and GmCMT3 (Table S4 in File S1). It has been suggested that the presence of multiple NLSs in proteins might modulate their level of import to the nucleus and hence control protein function [33].

### Evolutionary relationship among MTases in legumes

We studied the evolutionary relationship among the members of DNA MTase subfamilies. Full-length protein sequences of predicted plant MTases from eight plants species (five legumes, soybean, chickpea, *Medicago, Lotus* and pigeonpea along with grapevine, *Arabidopsis* and rice) were used for the construction of phylogenetic tree. The phylogenetic analysis corroborated the domain based classification of MTases into four subfamilies, grouping all the members into MET, CMT, DRM and DNMT2 subfamilies (Figure 2). MET and DRM subfamilies formed single clades, whereas CMT and DNMT2 subfamilies formed multiple clades. DNMT2 members were grouped into two separate clades. The closer inspection of sequences of the DNMT2 members in clade-II revealed absence of some conserved motifs (motifs IX and X) in them. These members might represent truncated annotated proteins (Figure S1D in File S1). CMT members were present in three clades (Figure 2). Similar grouping of CMTs in different clades have been reported in other studies as well. *AtCMT2* present in clade-III has been shown to carry out CHH methylation as opposed to CMTs present in other clades that catalyze CHG methylation [14]. Members of clade-III were conserved in all legumes analyzed except *Medicago* (namely *GmCMT4*, *CaCMT1*, *LjCMT4* and *CcCMT3*). Phylogenetic analysis suggests that some soybean MTase genes might represent duplicated gene pairs. This observation was confirmed by identification of *GmMET1* and *GmMET2*, *GmCMT2 and GmCMT3, GmDRM3 and GmDRM5, and GmDRM2 and GmDRM4* as paralogous genes from the SoyBase [34].

### Structural conservation of methyltransferase domain in legumes

In order to study the structural and functional conservation and/or unique features of legume MTases, we performed homology modeling of representative members of soybean and chickpea MTases employing multiple modeling approaches.

#### Structural features of CMT proteins

**Figure 3:**
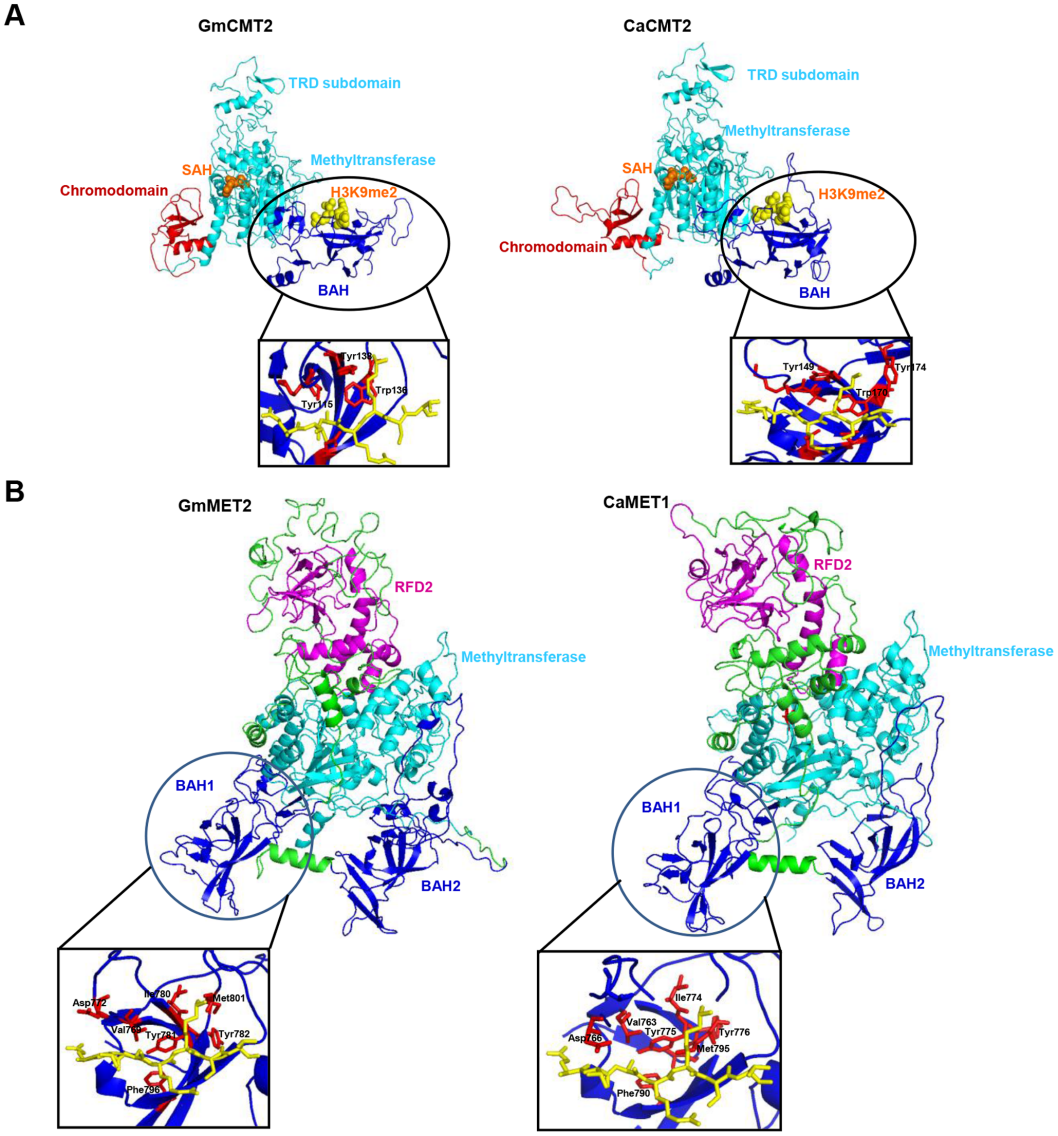
Three-dimensional (3D) structures of soybean and chickpea CMT and MET proteins constructed by homology modeling. (A) Ribbon representation of GmCMT2 and CaCMT2 protein structures with bound H3(1–32)K9me2 peptide. The BAH, methyltransferase, and Chr domains are colored in red, cyan, and blue, respectively, with bound S-adenosylhomocysteine (SAH) molecule (orange) and H3(1–32)K9me2 peptide (yellow, bound to BAH domain) shown in a space filling representation. *Inset* shows K9me2 accommodated within an aromatic cage formed by Tyr115, Tyr138 and Trp136 in GmCMT2 and by Tyr149, Tyr174 and Trp170 in CaCMT2. (B) Ribbon representation of homology modeled GmMET2 and CaMET1. The RFD, BAH and methyltransferase domains are colored in magenta, blue, and cyan, respectively. The K9me2 is accommodated within an aromatic cage in BAH1 of GmMET2 and in BAH1 of CaMET1 (*Inset*).

Recently, the crystal structure of a plant chromomethylase was solved, which provided important clues about the significance of each domain of MTases [35]. CMTs are unique to plants and are distinguished by the presence of a Chr domain between motifs I and IV within the methyltransferase domain (Figure 1; Figure S1B in File S1). Chr domains were first described in polycomb group proteins and implicated in targeting proteins to heterochromatin [36], [37]. As observed in the crystal structure of *Zea mays* ZmCMT3, GmCMT2 and CaCMT2 also formed similar 3D structures, with Chr domain looping out of the methyltransferase domain (Figure 3A). RMSD difference for the templates and the modeled structures ranged from 0.4–0.5%, which varied according to the identity of the target and template structure (Table S5 in File S1).

In plants, di-methylation of histone H3 at ninth lysine residue (H3K9me2) is mainly associated with heterochromatic regions and correlated with CHG methylation [38]. When modeled with H3(1–15)K9me2, the K9me2 side chain inserts into an aromatic cage in Chr domain, which is formed by Phe370, Trp397 and Tyr400 in GmCMT2, and Tyr415, Trp431 and Tyr434 in CaCMT2 (Figure S2 in File S1). When modeled with H3(1–32)K9me2, the K9me2 side chain was found to be inserted into an aromatic cage of BAH domain formed by Tyr115, Trp136 and Tyr138 in GmCMT2, and Tyr170, Trp172 and Tyr174 in CaCMT2 (Figure 3A) similar to Chr domain. The aromatic residues in the Chr and BAH domain were conserved in the other CMT members also, suggesting similar H3K9me2 binding to CMT members in legumes (Figure S1B in File S1).

#### Structural features of MET proteins

MET proteins share significant similarity with mammalian DNMT1 proteins. In order to gain insights into the functional significance of the domains present in MET1, we modeled MET of both soybean and chickpea using mouse DNMT1 crystal structure as template [39], [40]. To our knowledge, 3D structure of any plant MET protein has not been reported so far. Plant MET proteins have two RFD domains in comparison to one RFD domain in mammalian DNMT1. Therefore, we removed the N-terminal region up to first RFD domain from MET protein sequences in order to improve the alignment of target legume proteins with template mouse protein. The remaining sequence of MET proteins (GmMET2, 310-1555 aa and CaMET1, 326-1403 aa) were then modeled on DNMT1 structure (291-1620 aa). The two BAH domains present in METs were projected outwards in opposite direction relative to the methyltransferase domain (Figure 3B), and could serve as a platform for interaction with other proteins. The BAH1 domain of MET, composed of a twisted β-barrel with some extended segments, resembled BAH domain of CMT. The position of the BAH1 domain relative to its methyltransferase domain in MET proteins was also similar to that of the BAH domain of CMT relative to its methyltransferase domain, indicating a plausible similar function for the BAH domains of these two proteins. These results imply that the BAH1 domain of MET1 may recognize methylated-lysine marks using aromatic cage capture (Asp772, Ile780, Met801, Val769, Tyr781, Tyr782 and Phe796 in BAH1 of GmMET2 and Asp766, Ile774, Val763, Tyr775, Tyr776, Phe790 and Met795 in BAH1 of CaMET1) (Figure 3B) similar to that reported for mammalian DNMT1 [41]. In contrast, no aromatic cage forming residues were identified following alignment of the BAH2 domain of MET with BAH domain of CMT. BAH2 showed significant similarity with the polybromo BAH domain (24% identical as opposed to 14% of BAH1) and may be involved in state-specific interactions with histones and other chromatin proteins [41]. However, these speculations need to be substantiated experimentally. Since RFD domain of MET also seems to be interacting with the catalytic site through hydrogen bonds or non-bonded interactions, thus masking the catalytic center similar to that in DNMT1 (Figure S3A in File S1). Thus, RFD domain in plant METs may inhibit the binding of DNA to the catalytic motif of unmethylated CpG dinucleotides that emerge from the replication complex. In addition, comparison of MTase domain of CMT and MET suggested differences in the TRD subdomain in the regions further from catalytic center (arrow, Figure S3B in File S1). There are two-stranded antiparallel β sheets in CMT, whereas, a loop is present in MET, that may be interacting with the loop extending from BAH2, suggesting that both MTases might have a similar DNA recognition mode but different regulatory mechanisms.

#### Structural features of DRM and DNMT2 proteins

**Figure 4:**
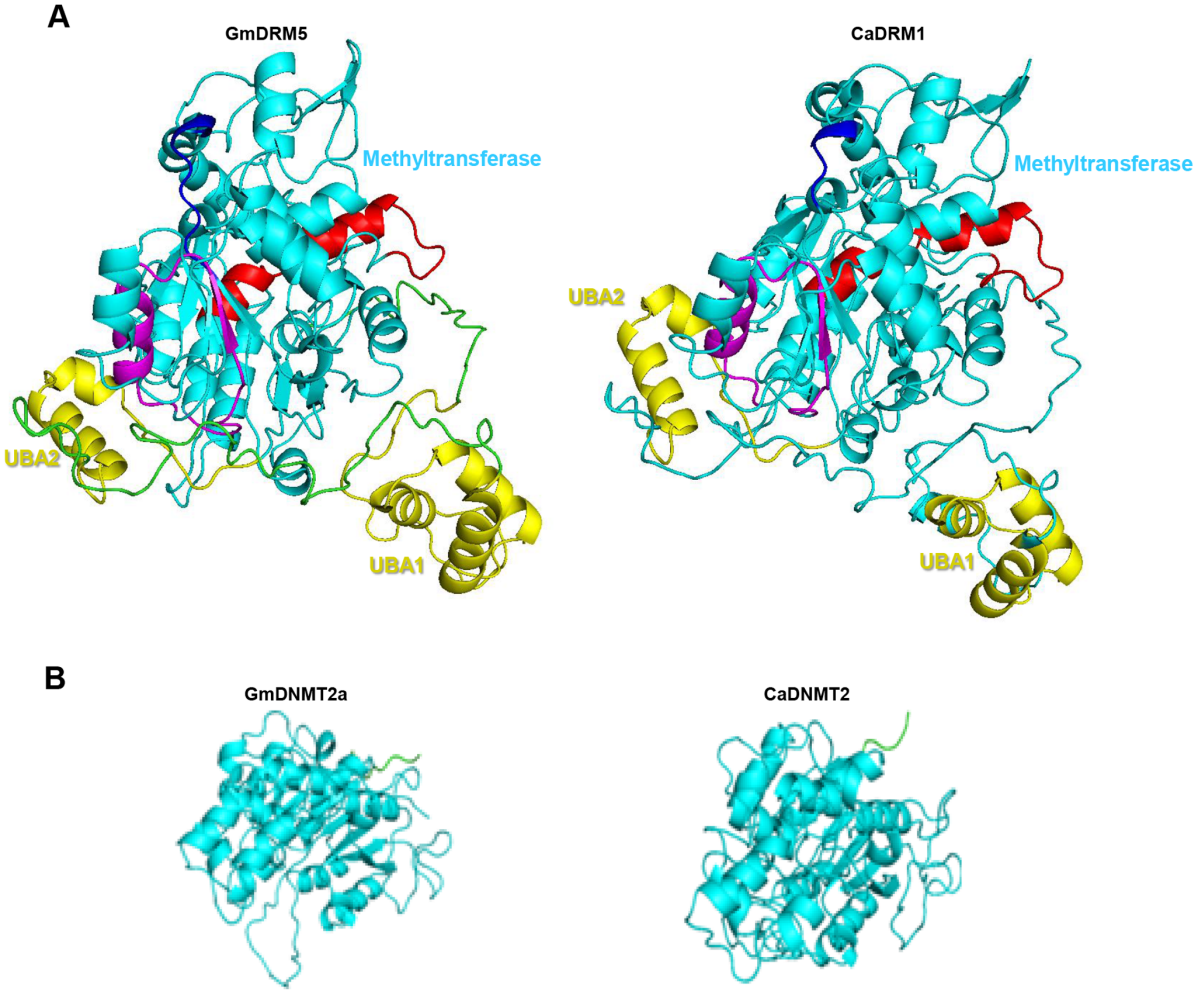
3D structures of modeled soybean and chickpea DRM and DNMT2 proteins. (A) Ribbon representation of modeled GmDRM2 and CaDRM2. The UBA and methyltransferase domains are colored in yellow and cyan, respectively. Motif I and motif X of methyltransferase domain are colored as magenta and red, respectively. (B) Ribbon representation of homology modeled GmDNMT2a and CaDNMT2. The methyltransferase domain is colored in cyan.

DRM proteins exist exclusively in plants. The structure of DRM proteins has not been elucidated so far. Therefore, we modeled DRM protein structure by threading using Phyre2. DRM has circular permutation of motifs in the methyltransferase domain [42]. Comparative analysis of the CMT3 and DRM structures suggested that circular permutation might allow the general methyltransferase domain structure to be maintained because motifs I (magenta color, Figure 4A) and X (red color, Figure 4A) that make the S-adenosyl L- methionine (SAM) binding site, are located in close proximity in the folded structure (Figure 4A, Figure S4A in File S1). The TRD region was also folded differently than CMT, suggesting distinct substrate specificity of DRMs. The UBA domain formed a compact three-helix bundle (Figure 4A and Figure S4B in File S1). The first loop contained a highly conserved MGF/MGY motif, which is required for correct folding and maintenance of UBA domain structure [32]. Comparison of the structures of UBA1, UBA2 and UBA3 revealed that all of these form very similar folds, except UBA2 in the proteins with three UBA domains (Figure S4B in File S1). The conserved large hydrophobic surface patch might be a common protein-interacting surface present in diverse UBA domains, such as in case of rice DRM2 interaction with the ATP-dependent RNA helicase eIF4A [43]. We also modeled DNMT2 class proteins and found conservation of MTase domain structure in these proteins (Figure 4B). Human DNMT2 (1G55) was used as template for modeling of soybean and chickpea DNMT2 proteins. The RMSD difference between the structures of template and target was very less (0.21), suggesting that these might also posses similar RNA methyltransferase (tRNA) like activity of mammalian DNMT2 [44], [45]. The conservation of cysteine and glutamate residues in motif IV and VI respectively, suggest that legume DNMT2 proteins might also follow DNA methyltransferase like mechanism to methylate their target tRNA. The models developed in this work will be a good starting point and serve as a valuable resource to understand the exact role of each domain.

### Differential gene expression of MTases during plant development

**Figure 5:**
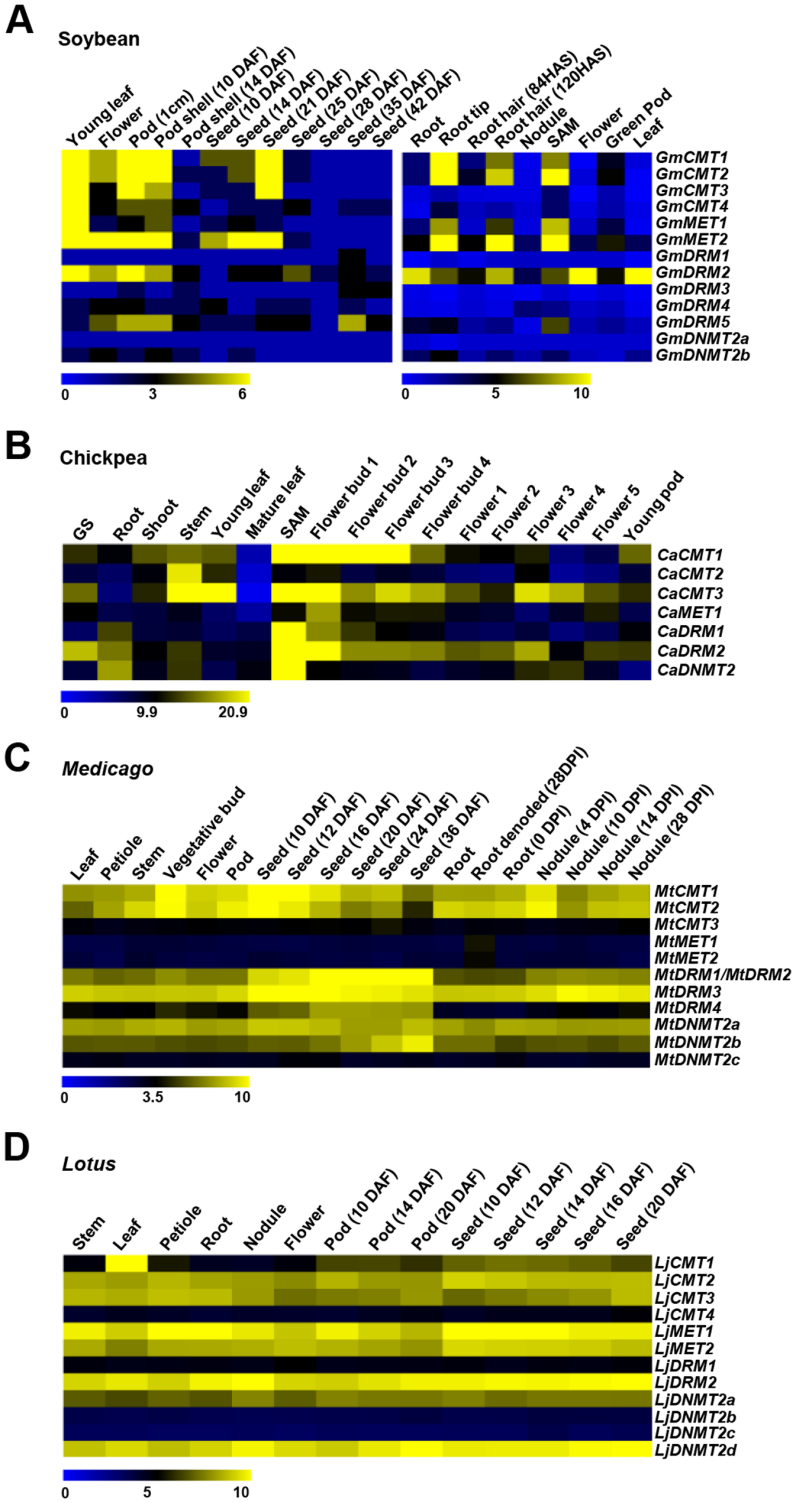
Expression profiles of MTase genes from soybean, chickpea, *Medicago* and *Lotus* in various tissues. Heatmaps showing expression of MTases in various tissues/organs and developmental stages (labeled on the top) of soybean (A), chickpea (B), *Medicago* (C) and *Lotus* (D). Color scale represents RPKM in case of soybean and chickpea members and log2 transformed signal intensities in case of *Lotus* and *Medicago*. GS, germinating seedling; DAF, days after fertilization; SAM, shoot apical meristem; HAS, hours after sowing; DPI, days post rhizobial inoculation.

MTases have been shown to play an important role in plant development. To gain insights into the putative function of MTases in legumes, we analyzed their spatial and temporal gene expression in various tissues/organs/developmental stages. For soybean, gene expression data from RNA seq experiments representing 21 tissues, including vegetative and seed development as well as nodule development stages [23], [46], was analyzed. Consistent with its role in DNA methylation maintenance, we found that soybean CMT was predominantly expressed in actively replicating cells in tissues, such as SAM, root tip, young leaf, young pod (with embryo) and seeds upto 25 days after fertilization, (DAF; Figure 5A). Among DRM members, *GmDRM1*, *GmDRM3* and *GmDRM4* were expressed at very low level in all the tissues analyzed. As *GmDRM2* and *GmDRM4*, and *GmDRM5* and *GmDRM3* represented duplicated genes, their expression profiles suggested neo- or non-functionalization of one of the paralogs.

**Figure 6:**
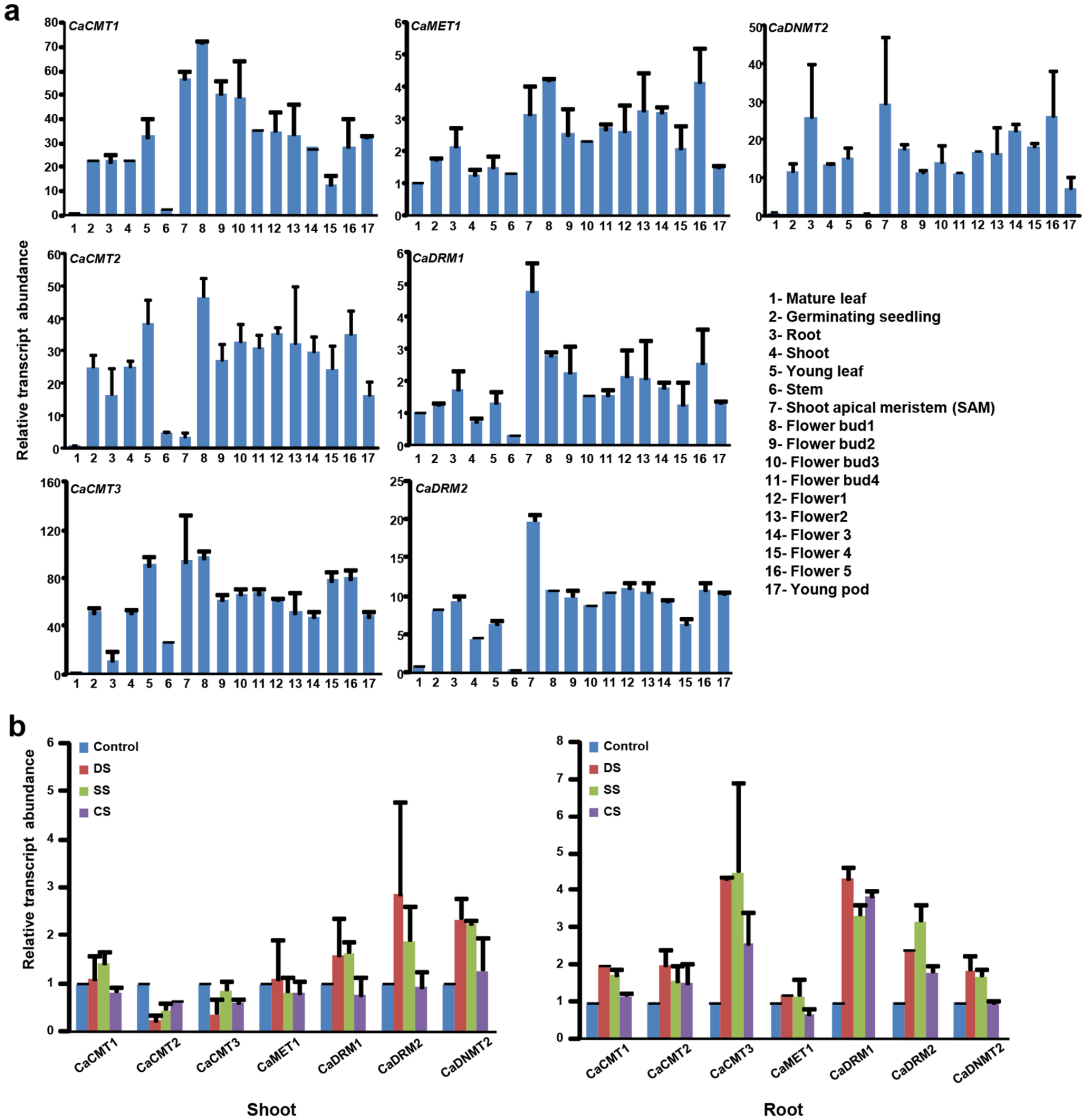
Real-time PCR analysis showing expression profiles of chickpea MTase genes in various tissues/developmental stages and under various abiotic stress conditions. (A) Validation of expression profiles of all chickpea MTase genes in various tissues/organs. Relative transcript level in different tissues is shown as compared to that of mature leaf for each gene. (B) Relative transcript levels in response to desiccation (DS), salt (SS) and cold stress (CS) as compared to control mock-treated root or shoot tissues are shown for each gene.

The expression of chickpea MTase genes was analyzed in 17 tissue samples (seven vegetative tissues/organs, nine stages of flower development and young pod) using RNA-seq data from our previous studies [18], [24]. *CaCMT2* exhibited highest expression in stem and its expression could not be detected during late stages of flower development. *CaCMT1* and *CaCMT3* showed higher expression in shoot apical meristem and initial stages of flower development (flower buds). Most of the chickpea MTases showed very low level of expression in mature leaf. All but *CaCMT2* and *CaMET1*, MTase genes in chickpea showed very high expression in SAM, which is comprised of actively dividing cells. *CaDRM2* showed higher expression in germinating seedling in addition to SAM and stages of flower development (Figure 5B). Interestingly, we found WUSATAg box in the promoters of *CaMET1* and *GmMET1,* as well as in *GmDRM3* and *GmDRM5*, which has been shown to be the target of WUSCHEL homeodomain proteins that regulate the formation and maintenance of shoot and root apical meristems [47]. Correlation of expression of *CaMET1*, *GmMET1* and *GmDRM5* in SAM, flower and pod suggests the role of this element in regulation of expression of these genes by WUSCHEL. The XYLAT element present in the promoters of the genes regulating secondary xylem development [48], was identified in the promoter of *CaDRM2*, *CaCMT3* and *GmCMT2* also, which is consistent with high expression of these genes in stem. We found fruit and embryo specific elements, such as ANAERO2CONSENSUS and CANBNNAPA in the promoter of *GmDRM2;* and CAATBOX1 and ANAERO2CONSENSUS in the promoter of *GmDRM5,* which correlated with their expression in flower and seed. Expression patterns of all the chickpea MTases in different tissues/organs and developmental stages was confirmed by real-time PCR analysis as well, which was in very good agreement with RNA-seq data (Figure 6A).

We also analyzed the expression of MTases in *Medicago* and *Lotus* using publicly available microarray datasets. In *Medicago*, expression of CMTs (*MtCMT1* and *MtCMT2*) increased during early stages of seed development and nodulation (Figure 5C). *MtDRM1* and *MtDRM2* exhibited very high expression during all stages of seed development with moderate expression during nodule development. *MtDRM3* exhibited very high expression during all stages of seed and nodule development. *MtDNMT2b* was found to be specifically expressed during late stages of seed development (20–36 DAF). In *Lotus*, *LjCMT1* showed leaf-specific expression; whereas *LjCMT2* and *LjCMT3* seemed to be involved in seed development and nodulation (Figure 5D). Both *LjMET1* and *LjMET2* were up-regulated during seed development with *LjMET1* having higher expression in vegetative tissues than *LjMET2*. Among DRM members *LjDRM1* was expressed at very low level, whereas *LjDRM2* exhibited very high expression in all the tissues analyzed. *LjDNMT2d* was expressed at very high levels in all the tissues analyzed as compared to other DNMT2 members.

### Differential gene expression of chickpea MTases under abiotic stresses

Further, we studied the expression patterns of MTases under various abiotic stress conditions (desiccation, salt and cold) in chickpea by real-time PCR analysis. We noted that expression of *CaDRM1* and *CaDRM2* was up-regulated under desiccation, cold and salt stress in chickpea roots. Although the expression of *CaMET1* remained unchanged in response to abiotic stresses in shoot, its transcript abundance was down-regulated in roots exposed to cold stress as compared to unstressed root control. *CaCMT3* was up-regulated in response to drought, salt and cold stress in roots in chickpea (Figure 6B). In contrast, *CaCMT2* was down-regulated in shoot under all the stress conditions analyzed. In addition, *CaDNMT2* was up-regulated in shoot following drought and salt stress treatments. Differential expression of CMT and DRM genes under specific stress conditions provides ample possibilities of differential regulation of plant processes by these MTases in response to abiotic stresses. This is consistent with the role of DRMs and CMTs to perpetuate asymmetric cytosine methylation patterns that might orchestrate differential gene expression in response to stress.

### Subcellular localization of chickpea MTase fusion proteins

**Figure 7:**
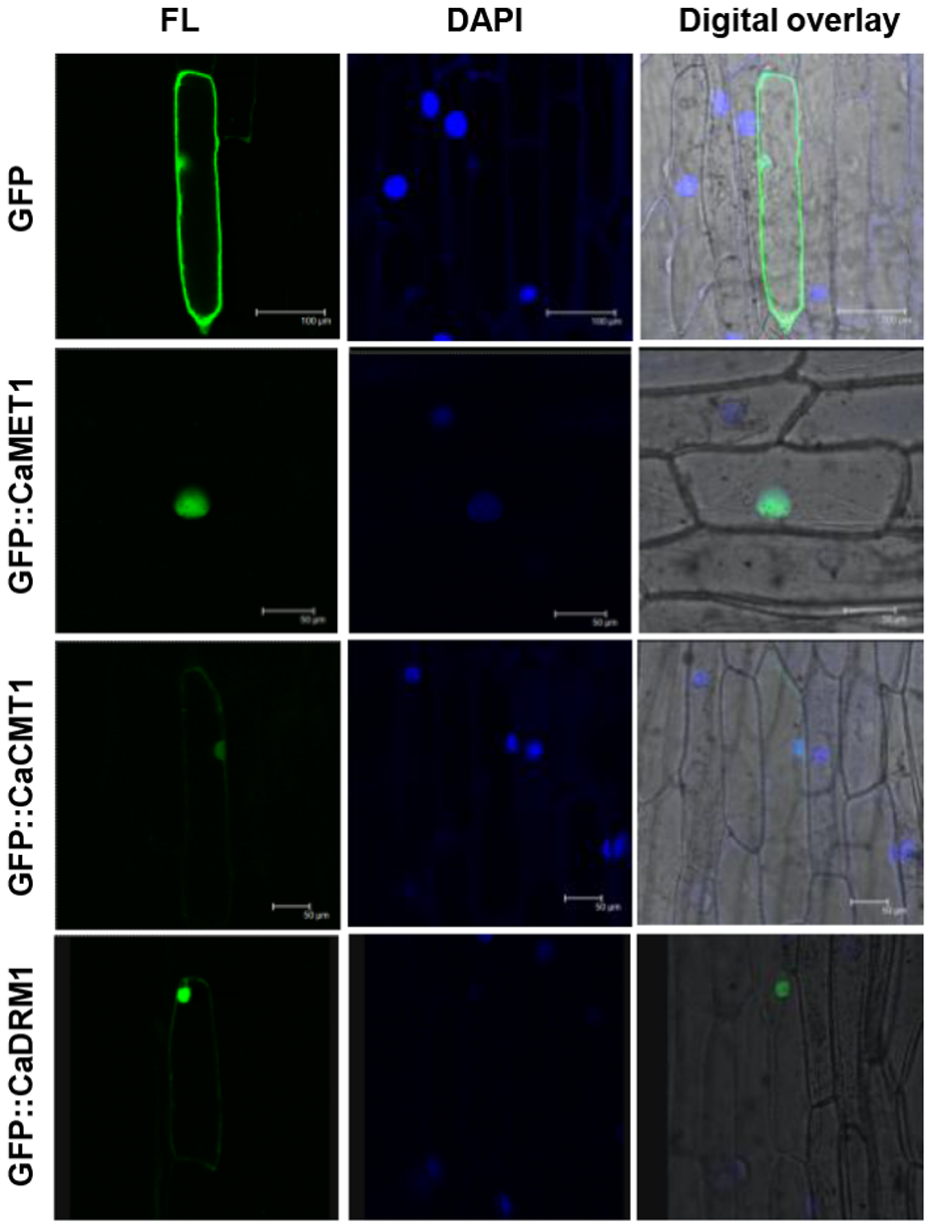
Subcellular localization of chickpea MTases fused with GFP. Visualization of GFP fused CaMET1, CaCMT1 and CaDRM1 as compared to full-length psGFPs in vector control (pUC based: 35S:psGFPs:tNos) in onion epidermal cells. FL, GFP fluorescence; DAPI, DAPI staining.

Tobacco and rice CMT and DRM proteins have been shown to be localized in nucleus [43], [49]. We also predicted NLSs in chickpea and soybean CMT and DRM proteins. To substantiate our prediction, we studied subcellular localization of chickpea MTases, CaCMT1, CaDRM1 and CaMET1 cloned in pUC based 35S-psGFP-tNOS vector with N-terminal GFP fusion. These fusion proteins were transiently expressed in onion epidermal cells. GFP-CaMET1, GFP-CaCMT1 and GFP-CaDRM1 were found to be localized specifically in the nucleus, whereas GFP-vector control was detected in whole cell (Figure 7). MET and CMT in *Arabidopsis* are known to be involved in the maintenance of methylation patterns during and after DNA replication, respectively. Localization of GFP fusion proteins of CaMET, CaCMT and CaDRM specifically in the nucleus, suggests the functional conservation of these MTases in legumes as well.

## Discussion

DNA methylation is an important epigenetic mark established by DNA MTases. MTases belong to four major subfamilies, MET, CMT, DRM and DNMT2 in plants [1]. So far, MTases have been analyzed in model plants, *Arabidopsis* and rice, only. In the present study, we identified MTases in five legumes and grouped them into four subfamilies based on domain organization and phylogenetic relationship. We identified upto four members of CMT, two members of MET, five DRMs and four DNMT2s in various legumes. These members could be clustered into different clades, which differ in domain/motif organization and methyltransferase activity in different sequence context. For example, clade-III of CMT has been recently shown to posses CHH methylation as opposed to CMTs in clade-I and clade-II that carries out CHG methylation [14]. Our detailed structural analysis highlights the residues conserved in Chr and BAH domains of CMTs and METs for recognition of specific methylated marks on histones. In addition, the comprehensive expression analyses of MTases in different legumes provide evidence for their diverse roles in various aspects of plant development and abiotic stress responses.

Chromomethylases are the plant-specific MTases, which have been most studied in plants. Our structure modeling analysis of chickpea and soybean CMTs (GmCMT2 and CaCMT2) suggested that both the chromo and BAH domains contain conserved aromatic residues that could form aromatic cage to recognize methylated histone (H3K9me2). These residues were conserved among other legume CMTs as well. The BAH and Chr domains could potentially increase the binding affinity of CMTs to methylated histones similar to *Z. mays* CMT3 [35].

MET family members are very similar to mammalian DNMT1 class [1]. Modeling of METs suggested that MET and DNMT1 might share similar structure and hence molecular interactions. RFD domain has been reported as an inhibitor of DNA binding to MTase domain in DNMT1 [40], [50], and this inhibition could be relieved by binding of Uhrf1 protein to DNMT1 in mammals [50], [51] and its homolog, Variant In Methylation (VIM) protein, to MET in plants [52]. Structural comparison suggested that autoinhibition mediated by RFD domain in DNMT1 might hold true for plant MET members also. In addition to RFD domain, the N-terminus of MET harbor two BAH domains, which might act as a site for protein-protein interactions. Our modeling effort identified one BAH domain (BAH1) similar to BAH domain of CMTs and it might be involved in interaction of MET with methylated histones tails, whereas other BAH domain (BAH2) might be involved in its interaction with other proteins (such as HDA6 and MEA) as reported previously [53], [54], providing a link between DNA replication, methylation and transcriptional regulation [41].

DRMs are present only in plants and members of DRM family could be identified in legumes too. The peculiarity of this family is a circular permutation of their conservative motifs, the motifs VI–X are followed by the motifs I-IV in the methyltransferase domain. Despite the availability of numerous crystal structures of MTases, none of the earlier studies reported the structure of a plant protein with a circularly-permutated motif order. The tertiary structural model of DRM MTase preserves a common fold of SAM-dependent MTases. The spatial location of most residues thought to be involved in formation of protein hydrophobic core, cofactor and substrate DNA binding and catalysis appears to be perfectly conserved despite a different topology of the protein backbone. The expression profiles of members of two DRM clades suggest that catalytically active members (*GmDRM2* and *GmDRM5*) are expressed at higher levels than inactive members (*GmDRM3* and *GmDRM4*). It would be important to investigate the relationship between DRM2 and DRM3 biochemically for in-depth understanding of the mechanism of *de novo* DNA methylation in plants.

DNMT2 genes are highly conserved across species, suggesting strong evolutionary selection pressure [55]. Recent findings have established DNMT2 as a tRNA methyltransferase that plays an important role under stress conditions [55], [56]. We also identified a total of 16 members of DNMT2 family in all the legumes lacking conserved N-terminal regulatory domain, but possess catalytic C-terminal domain. Increased expression of chickpea DNMT (*CaDNMT2*) under drought and salt stress in shoot suggested that DNMT2 might be involved in abiotic stress responses in chickpea as reported recently in Drosophila [57].

The comprehensive gene expression analyses of MTases in different legumes suggested their overlapping and specific roles during plant development. The higher gene expression of MET members in early stages of flower and seed development in soybean, chickpea and *Lotus* suggested their role in the maintenance of methylation during gametogenesis and embryogenesis in legumes. Similar expression patterns of MET gene have been observed in *Arabidopsis* and rice as well [10], [54], [58]–[60]. Interestingly, however, the expression of both MET genes in *Medicago* was very low in all the tissues/organs analyzed. Presumably, these genes might be expressed in specific tissues/cell-types not represented in this study. *GmCMT1* and *GmCMT2* exhibited similar expression patterns, but different than that of *GmCMT3* and *GmCMT4*. In chickpea also, the expression patterns of *CaCMT1*, *CaCMT2* and *CaCMT3* were quite different to each other. In *Medicago*, *MtCMT3* was expressed at very low level as compared to *MtCMT1* and *MtCMT2* in all the tissues. Likewise, *LjCMT4* was expressed at very low level as compared to other CMT members. Similar observations were made for DRM and DNMT2 subfamilies. Interestingly, lower expression of CMTs (*GmCMT1*, *GmCMT2*, *GmCMT4*, *MtCMT1*, *MtCMT2*, *LjCMT2*, and *LjCMT3*) and higher expression of DRMs (*GmDRM4*, *MtDRM1*, *MtDRM2*, *MtDRM3*, and *LjDRM2*) during nodule development suggested their plausible role in this legume-specific biological process. A few members of different subfamilies were expressed in tissue/developmental stage-specific manner as well. Altogether, these results suggested the sub- or pseudo-functionalization of members of a subfamily within a legume species. In addition, the distinct expression patterns of same subfamily genes in different legumes suggested that MTases might perform species-specific functions. Recently, epigenetic modifications have been shown to play crucial role in response to environmental stimuli [61], [62]. We also detected the differential regulation of transcript abundance of MTase genes under abiotic stress conditions in chickpea. In particular, higher transcript levels of CMT and DRM genes during abiotic stress suggested their involvement in stress-induced DNA methylation changes. It will be interesting to elucidate the precise role of MTases during development and/or abiotic stress response in different legumes.

In conclusion, we have identified members of MTases in legumes and deciphered unique structural features of each subfamily that could be attributed to specific domain organization in each subfamily. Differential transcript abundance of MTase genes in different tissues/organs highlighted their importance in regulating developmental processes, such as flower and seed development, and nodulation in legumes. Our study provides evidence for the role of MTases during environmental stress conditions as well. Altogether, this work bridges the knowledge of MTases in legumes and makes use of structural model for studies on protein functions that remain intractable in absence of a suitable structural model of MTases in higher plants.

## Supporting Information

File S1 The following supporting information is available in the online version of this article: **Table S1.** Details of known methyltransferases of *Arabidopsis* and rice. **Table S2.** List of primers used in this study. **Table S3.** Details of methyltransferases identified in three legumes and grapevine. **Table S4.** Nuclear localization signal predicted in chickpea and soybean methyltransferases. **Table S5.** Summary of homology modeling statistics of representative members of soybean and chickpea methyltransferases. **Figure S1. Multiple sequence alignment of all the classes of MTases identified in legumes.** Multiple sequence alignments were generated using JalView to highlight the conserved domains (line on top of alignments) and residues (black rectangular boxes) in MET (A), CMT (B), DRM (C) and DNMT2 (D). **Figure S2.** **Three-dimensional (3D) structures of soybean and chickpea CMT proteins constructed by homology modeling.** Ribbon representation of GmCMT2 and CaCMT2 protein structures with bound H3(1–15)K9me2 peptide. The BAH, methyltransferase, and Chr domains are colored in red, cyan, and blue, respectively, with bound S-adenosylhomocysteine (SAH) molecule (orange) and H3(1–15)K9me2 peptide (yellow, bound to Chr domain) shown in a space filling representation. The K9me2 is accommodated within an aromatic cage formed by Tyr400, Phe370 and Trp397in GmCMT2 and by Tyr415, Trp431 and Tyr434 in CaCMT2 (*Inset*). Intermolecular hydrogen bonds between H3K9me2 peptide and Chr domain are designated by dashed lines (*Inset*). **Figure S3. Structural features of plant METs.** (A) The RFD domain pluged into the DNA-binding pocket. The RFD domain (magenta) is positioned in the DNA-binding pocket (cyan) of MET and stabilized by several hydrogen bonds (inset; yellow dashed lines) or non-bonded interactions (inset; interacting residues shown as sticks) with the catalytic domain. PCQ region of the catalytic domain is highlighted in red color. (B) Comparison of the TRD subdomain of MET with CMT. Comparison of the TRD subdomains of MET (purple) and CMT (cyan) by superimposing their structures. The regulatory region of TRD subdomain in CMTs is occupied by two antiparallel sheets (arrow), whereas only loop is present in MET indicative of different regulatory mechanisms in these MTases. Chromodomain is shown in red color. **Figure S4. Structural features of plant DRMs.** (A) Ribbon representation of the structure of CMT with bound SAH to motif I (magenta) and motif X (red). Bound SAH molecule shown in a space filling representation. (B) The UBA domains (UBA1 and UBA2) are colored in cyan and green. (C) Comparison of UBA1 (cyan), UBA2 (green) and UBA3 (magenta) structures with conserved MGF/MGY shown as sticks.
(PDF)
