# Supplementary material for: Genomic Survey, Gene Expression Analysis and Structural Modeling Suggest Diverse Roles of DNA Methyltransferases in Legumes
Source: PLoS One. 2014 Feb 25;9(2):e88947. doi: 10.1371/journal.pone.0088947 (PMC3934875; doi:10.1371/journal.pone.0088947)

Table S1. Details of known methyltransferases of *Arabidopsis* and rice.

Gene_name	Gene identifier	Chromosome	Gene length (bp)	Protein length (aa)
Arabidopsis thaliana				
AtCMT1	AT1G80740	Chr1	4655	791
AtCMT2	AT4G19020	Chr4	6684	1295
AtCMT3	AT1G69770	Chr1	5268	839
AtMET2	AT4G14140	Chr4	5787	1545
AtMET3	AT4G13610	Chr4	6210	1404
AtMETIIb	AT4G08990	Chr4	5716	1512
AtMET4/AtDDM2	AT5G49160	Chr5	6140	1534
AtDRM1	AT5G15380	Chr5	3480	624
AtDRM2	AT5G14620	Chr5	3452	626
AtDRM3	AT3G17310	Chr3	4242	710
AtDNMT2	AT5G25480	Chr5	2390	383
Oryza sativa				
OsMET1	LOC_Os03g58400	Chr3	2245	376
OsMET2	LOC_Os07g08500	Chr7	2298	377
OsCMT1	LOC_Os03g12570	Chr3	11118	1787
OsCMT2	LOC_Os05g13780	Chr5	13681	1407
OsCMT3	LOC_Os10g01570	Chr10	5284	371
OsDRM1a	LOC_Os11g01810	Chr11	4216	473
OsDRM2	LOC_Os03g02010	Chr3	3700	507
OsDNMT2	LOC_Os01g42630	Chr1	2248	280

Table S2. List of primers used in this study.

**Primers for cloning in pUC based 35S-psGFPs-tNOS vector**	
**Gene name**	**Primer sequence**
*CaCMT1*_F	5’-ATAGGGCCCATGATCCAGAATG-3’
*CaCMT1*_R	5’-AATCCCGGGTCAAAGAATAGGA-3’
*CaDRM1*_F	5’-TTAGGGCCCATGGGTGATGTT-3’
*CaDRM1*_R	5’-AATCCCGGGTCTATAATTTGGTGA-3’
*CaMET1*_F	5’-AATGGGCCCATGTTCAAAACAG-3’
*CaMET1*_R	5’-ATACCCGGGTCATCTGTTTCTTG-3’
**Primers for quantitative real-time PCR**	
**Gene name**	**Primer sequence**
*CaCMT2*_F	5’-GGAGATCCGATTTCCGATGA-3’
*CaCMT2*_R	5’-GATGGCTTCTTTTCCTTTTCTTGA-3’
*CaCMT1*_F	5’-GTTGCAGCTGGGTGTTATGGT-3’
*CaCMT1*_R	5’-GGGTAGAACCTCGTCAGGATGA-3’
*CaCMT3*_F	5’-TGACAAGAGCAGAGCCTCACA-3’
*CaCMT3*_R	5’-TTTCACGTATGCTAAGAACTCGATCT-3’
*CaDNMT2*_F	5’-GTGCTGATGCATGGCTTCTTT-3’
*CaDNMT2*_R	5’-CCAGTGTCCTTCTGGAGACCTT-3’
*CaDRM1*_F	5’-CATGATGCATTTCCACTGTTGA-3’
*CaDRM1*_R	5’-TTCCAGTGACCGTTTGCAAA-3’
*CaDRM2*_F	5’-GGGTTTTTGATCTGGTCATTTATCA-3’
*CaDRM2*_R	5’-AAGCTGAGAGACTTCCAATGGTATG-3’
*CaMET1*_F	5’-CGCCCAAATAGAAGAATTACAGACTT-3’
*CaMET1*_R	5’-GTCCGGTGATAAACAAATCGTTAA-3’
*Ca-EF1α*_F	5’-TCCACCACTTGGTCGTTTTG-3’
*Ca-EF1α*_R	5’-CTTAATGACACCGACAGCAACAG-3’

Table S3. Details of methyltransferases identified in three legumes and grapevine.

Gene_name	Gene identifier	Chromosome	Gene length (bp)	Protein length (aa)
Medicago truncatula				
MtCMT1	Medtr5g023660.1	Chr5	5816	835
MtCMT2	Medtr5g066780.1	Chr5	2810	800
MtCMT3	AC233109_38.1	-	5550	827
MtMET1	Medtr4g035150.1	Chr4	8048	1641
MtMET2	Medtr6g065580.1	Chr6	9053	1573
MtDRM1	Medtr3g011150.1	Chr3	6403	628
MtDRM2	Medtr5g019420.1	Chr5	8781	751
MtDRM3	Medtr6g005580.1	Chr6	9837	588
MtDRM4	AC235665_42.1	-	6403	628
MtDNMT2a	Medtr2g009140.1	Chr2	4840	378
MtDNMT2b	Medtr5g066830.1	Chr5	579	192
MtDNMT2c	Medtr8g062560.1	Chr8	935	151
Lotus japonicus				
LjCMT1	chr2.CM0065.100.nd	Chr2	7880	866
LjCMT2	chr4.CM0680.70.nc	Chr4	8536	900
LjCMT3	LjT31J09.230.nd	-	9005	536
LjCMT4	LjSGA_028177.1	-	1805	774
LjMET1	chr2.CM0272.410.nd	Chr2	3618	416
LjMET2	chr3.LjB23H05.80.nd	Chr3	6927	1534
LjDRM1	chr2.CM0008.250.nc	Chr2	3243	440
LjDRM2	LjB15A13.10.nd		9940	606
LjDNMT2a	chr6.CM0055.800.nd	Chr6	1516	328
LjDNMT2b	LjSGA_133586.1	-	449	147
LjDNMT2c	LjSGA_015229.1	-	1824	138
LjDNMT2d	LjSGA_084646.1	-	324	107
Cajanus cajan				
CcCMT1	C.cajan_12460	Chr6	5744	822
CcCMT2	C.cajan_15419	Chr8	5748	723
CcCMT3	C.cajan_04198	Chr11	18095	893
CcMET1	C.cajan_32735	Scaffold	6394	1290
CcDRM1	C.cajan_12070	Chr6	2128	411
CcDNMT2a	C.cajan_17086	Chr8	4156	384
CcDNMT2b	C.cajan_28099	Scaffold	1796	344
Vitis vinifera				
VvCMT1	GSVIVT01022685001	Chr2	32565	729
VvCMT2	GSVIVT01025386001	Chr6	13286	821
VvCMT3	GSVIVT01033464001	Chr8	5897	827
VvCMT4	GSVIVT01024446001	Chr16	42296	847
VvMET1	GSVIVT01000262001	Chr7	6078	1417
VvMET2	GSVIVT01000263001	Chr7	9060	1429
VvDRM1	GSVIVT01032445001	Chr14	13086	512
VvDNMT2a	GSVIVT01035808001	Chr4	4998	366
VvDNMT2b	GSVIVT01023152001	Chr12	6707	460
VvDNMT2c	GSVIVT01023383001	Chr12	294	74
VvDNMT2d	GSVIVT01022824001	Chr12	2035	83
VvDNMT2e	GSVIVT01023370001	Chr12	1908	60

Table S4. Nuclear localization signal predicted in chickpea and soybean methyltransferases.

Protein name	Position (aa)	NLS sequence	Score	Monopartite/ bipartitite
CaCMT1	93	GNPPKKKYKTS	9	monopartite
CaCMT2	187	EPKRVFFSEVQDDNPLDCLVEKLNIARLP	4.3	bipartite
	221	DAKKKVIPPCDYYCDTLYLLPYSTFVKLPSES	4.4	bipartite
CaCMT3	25	LPSQRKKAKHS	8.5	monopartite
	6	FSMGKRAQQSPQNDDVVSLLPSQRKKAKHS	7.3	bipartite
CaMET1	30	NKKKRNSSESSEQPAPTRKMPKRS	11.8	bipartite
CaDRM1	325	RKRGYIHNLPIENRYPLLPLPPRTIHDA	6	bipartite
CaDRM2	305	DRNKRPKYEY	5.5	monopartite
CaDNMT2	285	VYPDSKRCCCFTKSY	6	monopartite
GmCMT1	8	PSKRKTRSSASPAAAPPSKRASRS	9.4	bipartite
GmCMT2	28	LPSQRKKPKHS	9.5	monopartite
	9	KKRSASDATDDAQNDTVSLLPSQRKKPK	7	bipartite
GmCMT3	53	RYATMAKRKTSDASDAQDDTVSLLPSQRNKAKHP	6.2	bipartite
	204	DLVDKKRIFISDVKDVNPLDCIVSKVKIYKKS	6.6	bipartite
GmCMT4	340	NRFRNCASPLDDERNRQIVIFMDMVKFLKP	6.1	bipartite
GmMET1	43	DKQKKRSLSESSEQTAAMRKMPKR	10	bipartite
GmMET2	43	DKQKKRSLLESSEQPAATRKMPKR	9.6	bipartite
GmDRM1	264	RKRGYIHNLPTHNRSPLLPIPPLTIQEA	5.3	bipartite
GmDRM2	212	KSNKRRLYDYEVLGRKKPRG	13.7	bipartite
GmDRM3	330	GRGKRPKYE	7	monopartite
GmDRM4	365	GRGKRPKYE	7	monopartite
GmDRM5	201	KSNKRRFYDYEVLGRKKPRG	12.3	bipartite
GmDNMT2a	96	RPRYFCLAKRKPSSFVNECLNNLLIQSSPPLFGHFD	4.4	bipartite
GmDNMT2b	295	VYPDSKRCCCFTKSY	6	bipartite

Table S5. Summary of homology modeling statistics of representative members of soybean and chickpea methyltransferases.

**Protein name**	**Template used for homology modelling (PDB ID)**	**Identity (%)**	**Query Coverage (%)**	**Residues in favoured region (%)**	**Residues in allowed regions (%)**	**Residues in outlier regions (%)**	**RMSD (Å)**
**CaCMT2**	4FT2	53	90	86.1	7.9	6.0	0.549
**CaCMT2**	4FT4	53	90	88.9	6.6	4.5	0.905
**CaMET1**	3AV4	42	84	90.1	6.6	3.4	3.09
**CaDRM1**	GmDRM5	81	100	86.4	6.6	7.0	0.184
**CaDNMT2**	1G55	37	97	90.2	6.9	2.9	0.211
**GmCMT2**	4FT2	55	94	84.5	10.5	5.0	0.417
**GmCMT2**	4FT4	55	94	88.7	7.8	3.5	0.603
**GmMET2**	3AV4	31	93	89.7	6.8	3.5	3.43
**GmDRM5**	Threading[1]	-	-	86.5	5.4	8.1	-
**GmDNMT2a**	1G55	38	96	90.3	6.3	3.4	0.210

[1]78% residues modeled with more than 90% confidence.

Figure S1. **Multiple sequence alignment of all the classes of MTases identified in legumes.** Multiple sequence alignments were generated using JalView to highlight the conserved domains (line on top of alignments) and residues (black rectangular boxes) in MET (A), CMT (B), DRM (C) and DNMT2 (D).

(A) MET

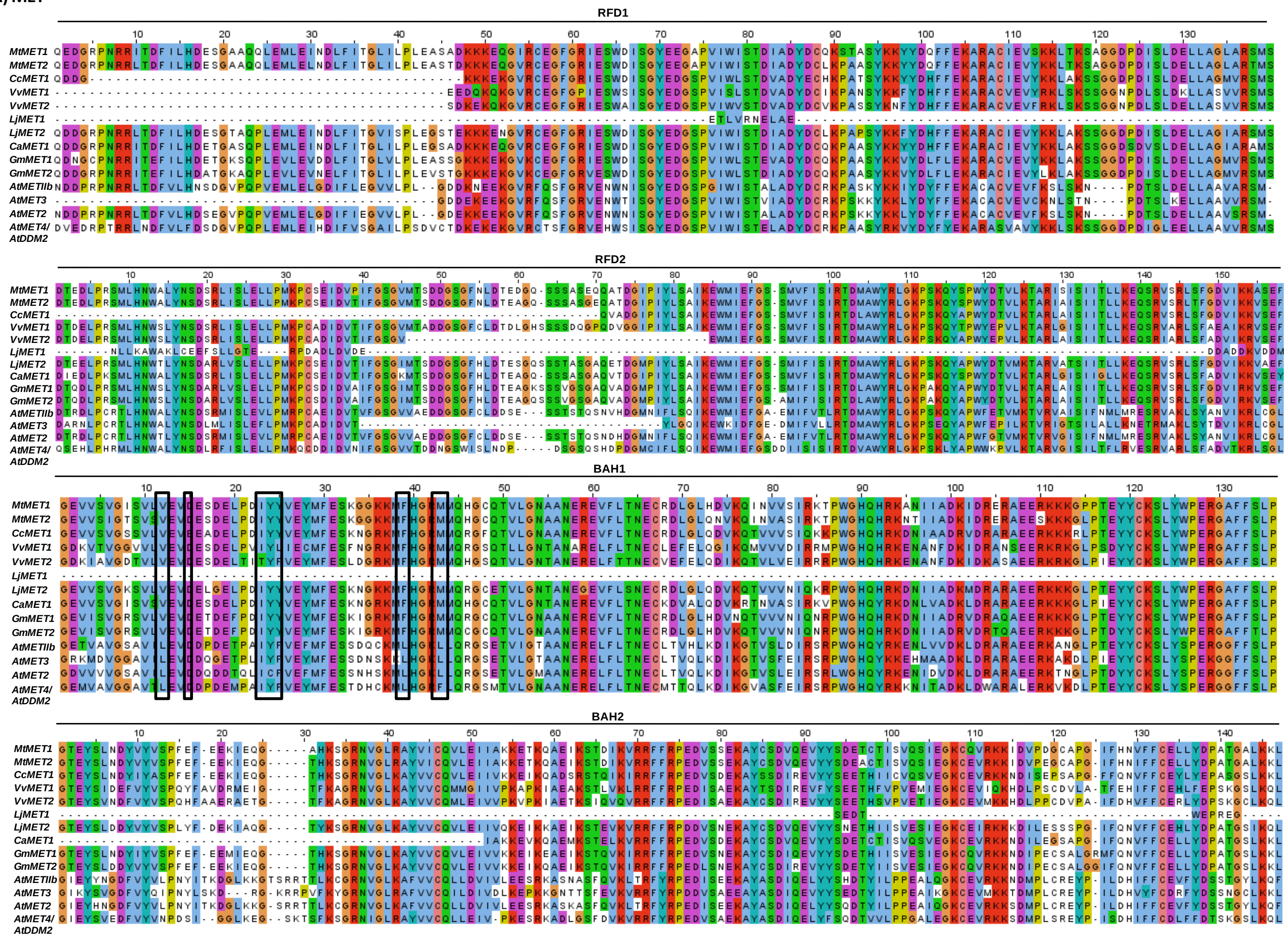

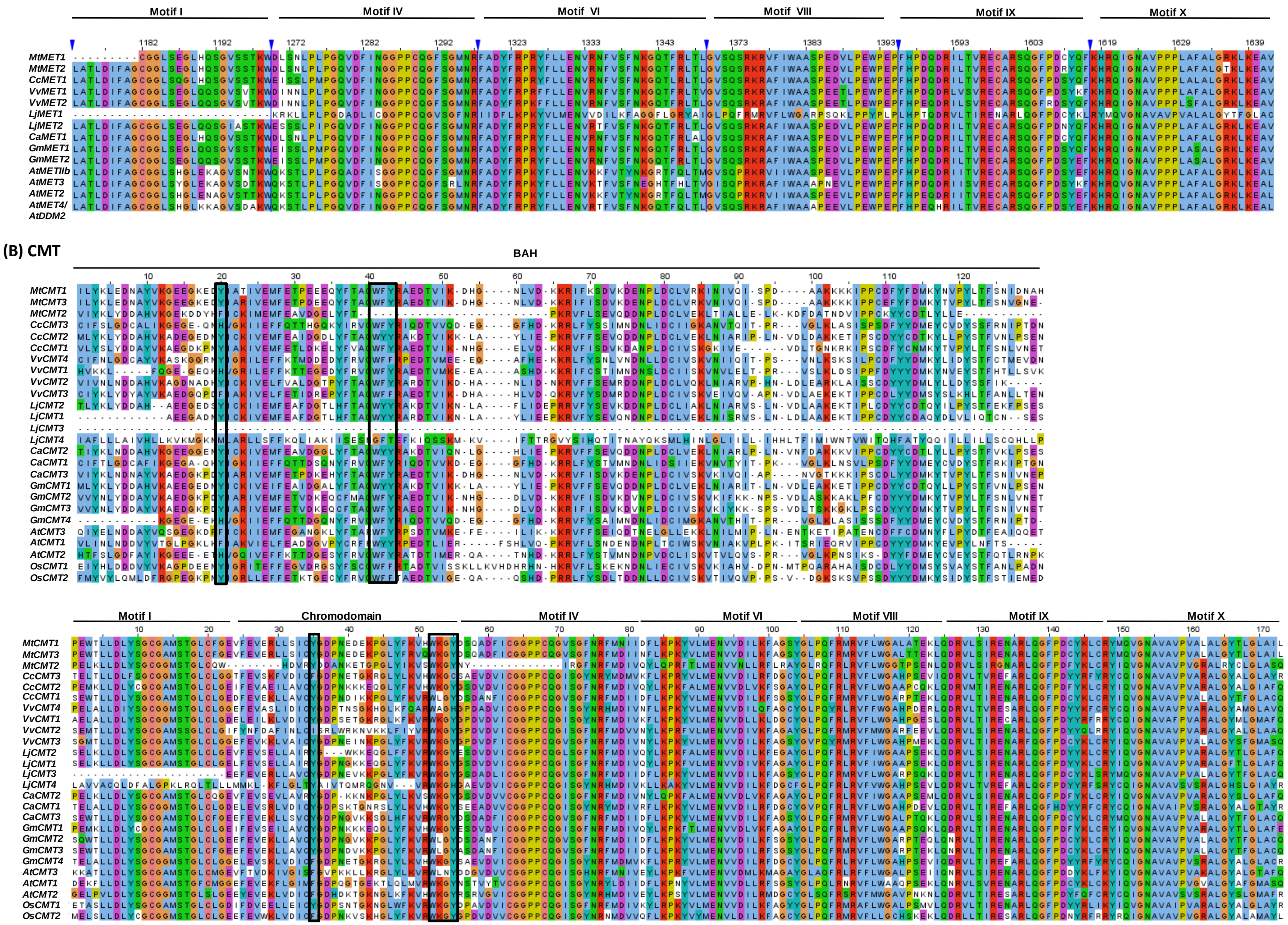
Motif I
Motif IV
Motif VI
Motif VIII
Motif IX
Motif X
MtMET1
MtMET2
CcMET1
VvMET1
VvMET2
LjMET1
LjMET2
CaMET1
GmMET1
GmMET2
AtMETIIb
AtMET3
AtMET2
AtMET4/
AtDDM2
(B) CMT
BAH
MtCMT1
MtCMT3
MtCMT2
CcCMT3
CcCMT2
CcCMT1
VvCMT4
VvCMT1
VvCMT2
VvCMT3
LjCMT2
LjCMT1
LjCMT3
LjCMT4
CaCMT2
CaCMT1
CaCMT3
GmCMT1
GmCMT2
GmCMT3
GmCMT4
AtCMT3
AtCMT1
AtCMT2
OsCMT1
OsCMT2
Chromodomain

(C) DRM

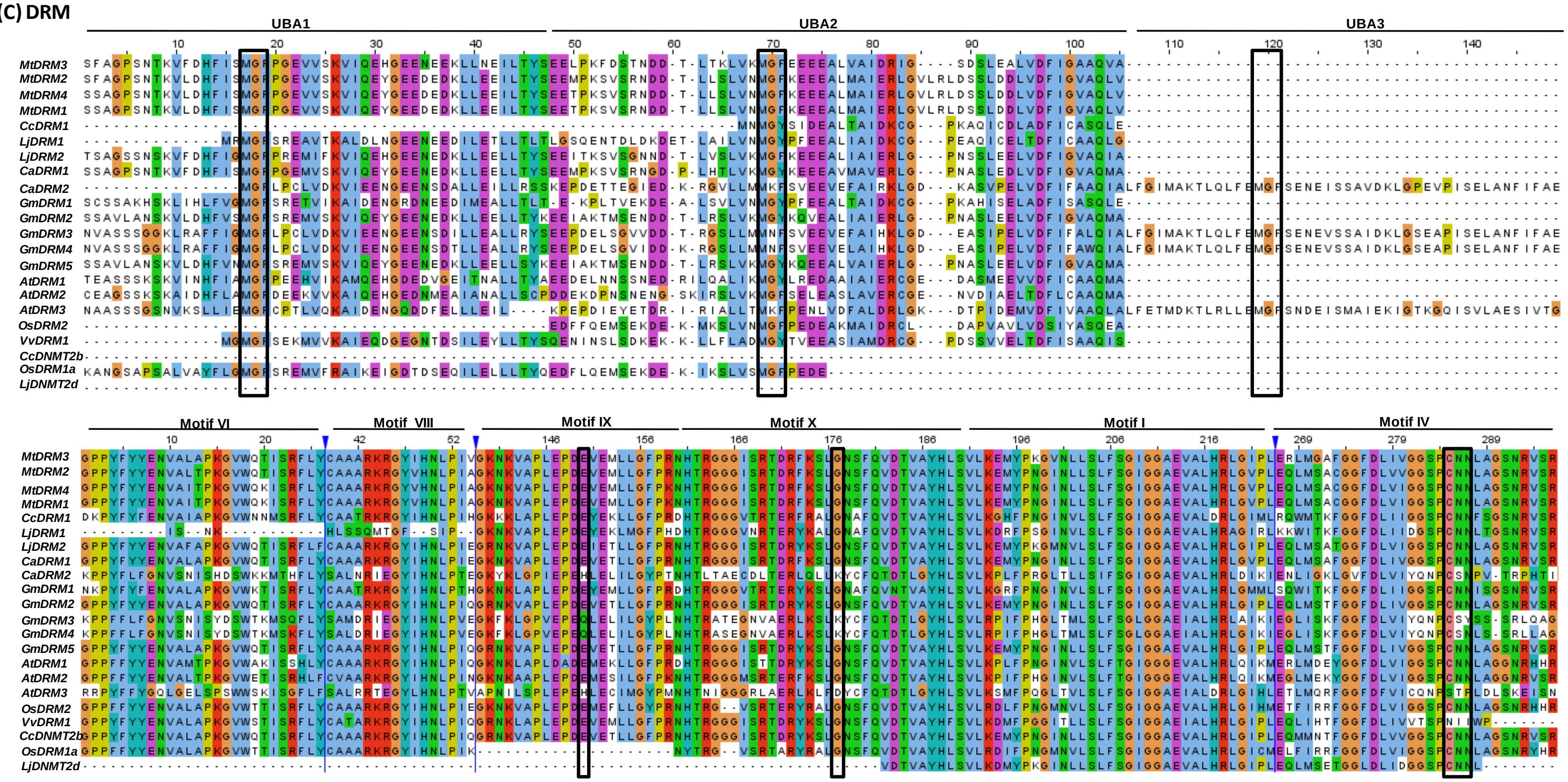
UBA1
UBA2
UBA3
Motif VI
Motif VIII
Motif IX
Motif X
Motif I
Motif IV
MtDRM3
MtDRM2
MtDRM4
MtDRM1
CcDRM1
LjDRM1
LjDRM2
CaDRM1
CaDRM2
GmDRM1
GmDRM2
GmDRM3
GmDRM4
GmDRM5
AtDRM1
AtDRM2
AtDRM3
OsDRM2
VvDRM1
CcDNMT2b
OsDRM1a
LjDNMT2d

(D) DNMT2

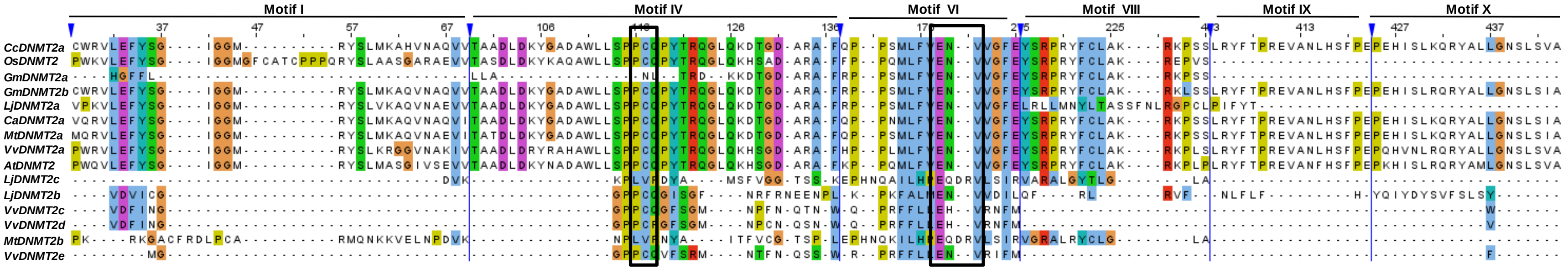
Motif I
Motif IV
Motif VI
Motif VIII
Motif IX
Motif X
CcDNMT2a
OsDNMT2
GmDNMT2a
GmDNMT2b
LjDNMT2a
CaDNMT2a
MtDNMT2a
VvDNMT2a
AtDNMT2
LjDNMT2c
LjDNMT2b
VvDNMT2c
VvDNMT2d
MtDNMT2b
VvDNMT2e

Figure S2. **Three-dimensional (3D) structures of soybean and chickpea CMT proteins constructed by homology modeling.** Ribbon representation of GmCMT2 and CaCMT2 protein structures with bound H3(1–15)K9me2 peptide. The BAH, methyltransferase, and Chr domains are colored in red, cyan, and blue, respectively, with bound S-adenosylhomocysteine (SAH) molecule (orange) and H3(1–15)K9me2 peptide (yellow, bound to Chr domain) shown in a space filling representation. The K9me2 is accommodated within an aromatic cage formed by Tyr400, Phe370 and Trp397in GmCMT2 and by Tyr415, Trp431 and Tyr434 in CaCMT2 (*Inset*). Intermolecular hydrogen bonds between H3K9me2 peptide and Chr domain are designated by dashed lines (*Inset*).

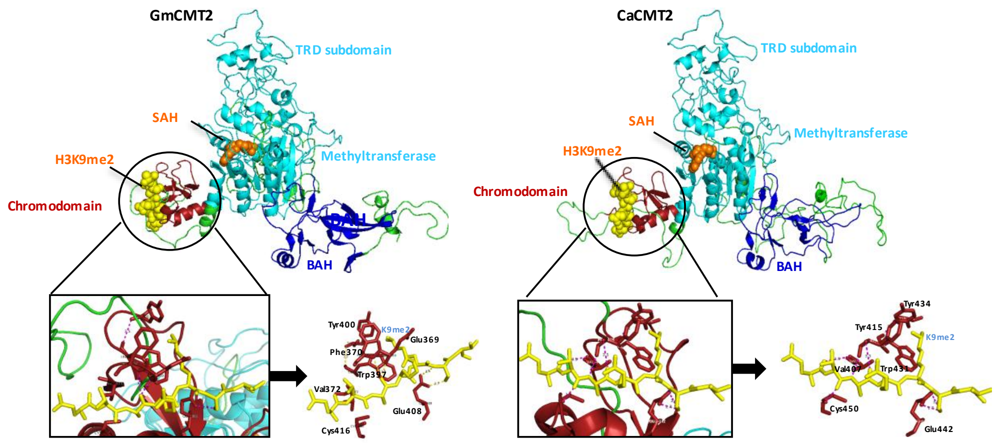

Figure S3. **Structural features of plant METs.** (A) The RFD domain pluged into the DNA-binding pocket. The RFD domain (magenta) is positioned in the DNA-binding pocket (cyan) of MET and stabilized by several hydrogen bonds (inset; yellow dashed lines) or non-bonded interactions (inset; interacting residues shown as sticks) with the catalytic domain. PCQ region of the catalytic domain is highlighted in red color. (B) Comparison of the TRD subdomain of MET with CMT. Comparison of the TRD subdomains of MET (purple) and CMT (cyan) by superimposing their structures. The regulatory region of TRD subdomain in CMTs is occupied by two antiparallel sheets (arrow), whereas only loop is present in MET indicative of different regulatory mechanisms in these MTases. Chromodomain is shown in red color.

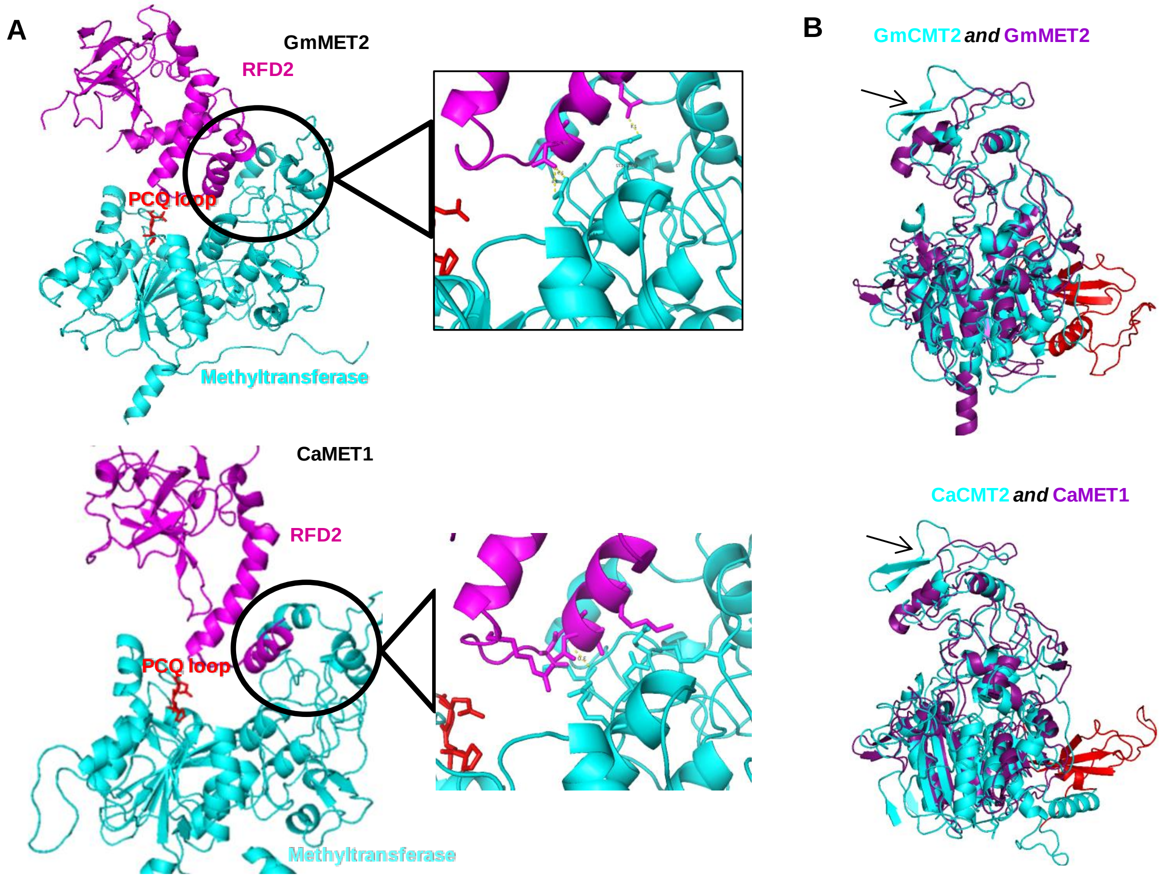

Figure S4. Structural features of plant DRMs. (A) Ribbon representation of the structure of CMT with bound SAH to motif I (magenta) and motif X (red). Bound SAH molecule shown in a space filling representation. (B) The UBA domains (UBA1 and UBA2) are colored in cyan and green. (C) Comparison of UBA1 (cyan), UBA2 (green) and UBA3 (magenta) structures with conserved MGF/MGY shown as sticks.

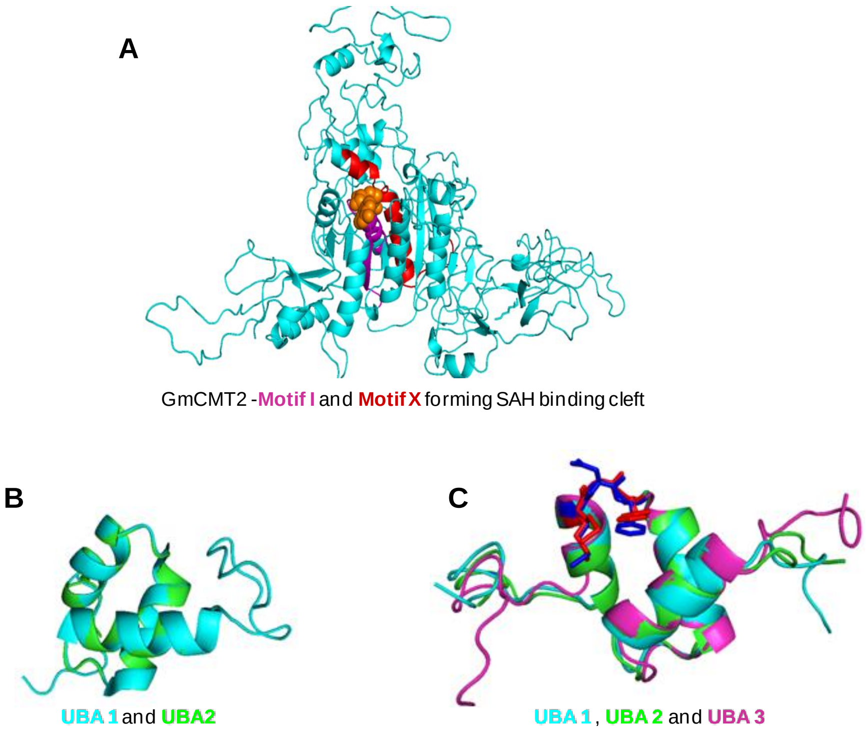

Supplement: File S1 — The following supporting information is available in the online version of this article: Table S1. Details of known methyltransferases of Arabidopsis and rice. Table S2. List of primers used in this study. Table S3. Details of methyltransferases identified in three legumes and grapevine. Table S4. Nuclear localization signal predicted in chickpea and soybean methyltransferases. Table S5. Summary of homology modeling statistics of representative members of soybean and chickpea methyltransferases. Figure S1. Multiple sequence alignment of all the classes of MTases identified in legumes. Multiple sequence alignments were generated using JalView to highlight the conserved domains (line on top of alignments) and residues (black rectangular boxes) in MET (A), CMT (B), DRM (C) and DNMT2 (D). Figure S2. Three-dimensional (3D) structures of soybean and chickpea CMT proteins constructed by homology modeling. Ribbon representation of GmCMT2 and CaCMT2 protein structures with bound H3(1–15)K9me2 peptide. The BAH, methyltransferase, and Chr domains are colored in red, cyan, and blue, respectively, with bound S-adenosylhomocysteine (SAH) molecule (orange) and H3(1–15)K9me2 peptide (yellow, bound to Chr domain) shown in a space filling representation. The K9me2 is accommodated within an aromatic cage formed by Tyr400, Phe370 and Trp397in GmCMT2 and by Tyr415, Trp431 and Tyr434 in CaCMT2 (Inset). Intermolecular hydrogen bonds between H3K9me2 peptide and Chr domain are designated by dashed lines (Inset). Figure S3. Structural features of plant METs. (A) The RFD domain pluged into the DNA-binding pocket. The RFD domain (magenta) is positioned in the DNA-binding pocket (cyan) of MET and stabilized by several hydrogen bonds (inset; yellow dashed lines) or non-bonded interactions (inset; interacting residues shown as sticks) with the catalytic domain. PCQ region of the catalytic domain is highlighted in red color. (B) Comparison of the TRD subdomain of MET with CMT. Comparison of the TR [file pone.0088947.s001.pdf]
